# Commentary: The Impact of Asking Intention or Self-Prediction Questions on Subsequent Behavior: A Meta-Analysis

**DOI:** 10.3389/fpsyg.2016.00879

**Published:** 2016-06-09

**Authors:** Angela M. Rodrigues, David P. French, Falko F. Sniehotta

**Affiliations:** ^1^Fuse, The UK Clinical Research Collaboration Centre for Excellence for Translational Research in Public Health, Newcastle UniversityNewcastle, UK; ^2^School of Psychological Sciences, Manchester UniversityManchester, UK

**Keywords:** question-behavior effect, mere-measurement effect, health behavior, behavior change, public health

The term question-behavior effect (QBE) refers to the idea that asking individuals about their beliefs and intentions regarding a particular behavior may change that behavior. Two recent systematic reviews (Rodrigues et al., 2015; Wood et al., 2015) set out to assess whether the QBE was a viable intervention for social policy and public health, i.e., can behavior change be brought about just by asking questions? The reviews came to different conclusions. This commentary aims to highlight commonalities and differences between the two reviews and concludes that the case for QBE in public health is currently limited.

## Inclusion criteria

The Wood review has taken an exploratory approach and included 116 observations of both randomized and non-randomized controlled studies in their meta-analyses examining effects of QBE on a wide range of behaviors. A subset of 37 comparisons tested for QBE on health behaviors (Wood et al., 2015). The Rodrigues review included 33 randomized controlled studies in the main meta-analysis on heath behavior (Rodrigues et al., 2015). One of the key differences between the two reviews relates to the inclusion of non-randomized studies in the Woods review. The Rodrigues review only included trials that randomly assigned participants to conditions testing QBE on health behaviors^1^. Randomization provides an opportunity to diminish the chance of confounding and problems with respect to differences between people in experimental groups (selection bias). The Cochrane Handbook (Higgins and Green, 2011) states that if non-randomized studies have low risk of bias, and if groups to be compared are homogeneous, then they could be considered for inclusion in meta-analysis.

## Behavioral domain classification

The classification of health behavior might also explain why studies and comparisons differ between these two reviews (Rodrigues et al., 2015; Wood et al., 2015). The Wood review classified behavior as health, prosocial, consumer, undesirable/risky behavior, and “other,” resulting in some studies included by the Rodrigues review being classified in other behavioral domains (i.e., prosocial, *k* = 3; Cioffi and Garner, 1998; Godin et al., 2008, 2010) and risky behaviors (*k* = 1; Levav and Fitzsimons, 2006).

## Risk of bias of primary studies

Quality appraisal of primary studies is a key requirement in systematic reviews (Moher et al., 2009). Widely accepted guidance for assessing risk of bias is available (Tacconelli, 2010; Higgins and Green, 2011). The Wood review did not include a standard assessment of the risk of bias. This lack of transparency regarding the risk of bias of the studies pooled to obtain an overall estimate of effect can be thought of as a level of uncertainty; we just cannot quantify how uncertain we are. Three sources of bias are particularly likely to have significant implications on QBE. Firstly, selective reporting of outcomes can lead to only significant QBE being reported; Secondly, not blinding participants can be problematic as knowledge of allocation might affect question elaboration or desirability bias in self-reported outcomes; and thirdly, incomplete outcome data not appropriately addressed might introduce risk as loss to follow-up could be different in study arms.

Despite finding considerable risk of bias, the Rodrigues review found that it did not substantially affect QBEs. Given that the Wood review included non-randomized studies, it is likely that the risk of bias is *at least* as high as in the Rodrigues review. However as sources of bias differ in non-randomized studies, the effects found in the Wood review might have been inflated through methodological bias in the included studies.

## Publication bias

Publication bias is an essential issue in this area, as allocating subgroups to QBE manipulations is often nested as sub-studies within other studies of more substantive interest, and hence not reported when non-significant (French and Sutton, 2010).

The Wood review acknowledged the importance of publication bias and concluded that publication bias was modest. A trim and fill analysis that showed a smaller, but still significant QBE. Likewise, the Rodrigues review concluded that there was evidence of publications bias. Despite the methodological differences, both reviews suggested that the QBE was inflated, at least slightly, as a result of publication bias.

The only safeguard against publication bias is to pre-register trials, a custom required by leading journals to consider RCTs for publication. There is evidence that generally trials confirming the authors' research hypothesis are more likely to be published (Hopewell et al., 2009) making it difficult to quantify the magnitude of the publication bias. A lack of pre-registration leaves the door open for data fishing exercises to obtain statistically significant results. The Rodrigues review identified only one pre-registered RCT in their review (Moreira et al., 2012) and, incidentally, this trial did not find evidence for a QBE. The Rodrigues review concluded that journals should abstain from publishing unregistered QBE studies. Two recent, large trials demonstrated the importance of registration as no evidence of QBE on uptake of colorectal cancer screening (O'Carroll et al., 2015) and for organ donation (O'Carroll et al., 2016) was found.

## Effect sizes

The Wood review found a heterogeneous, significant QBE on health behavior (*d* = 0.29 [0.19, 0.40]; *I*^2^ = 71%) and prosocial behavior (*d* = 0.19 [0.08, 030]); but no significant QBE on risky behaviors (*d* = −0.05 [−0.23, 0.13]). In contrast, the Rodrigues review found an overall smaller and heterogeneous (*I*^2^ = 44%) but still significant QBE on health behavior (*d* = 0.09 [0.04, 0.13]). To test whether the behavioral classification, which might be considered a subjective element of any review, could explain the differences in effect size, a new meta-analysis of the studies included by the Rodrigues review was undertaken. The new analysis revealed a similar trend to that found by Woods, albeit with differences in magnitude: (a) health behavior (*d* = 0.14 [0.07, 020]); (b) prosocial behavior (*d* = 0.05 [0.00, 0.10]); and (c) risky behaviors (*d* = 0.004 [−0.14, 0.15]). These analyses corroborate the argument that the choice of behavioral classification explains partially the differences in magnitude between the reviews; it is, however, only one part of the puzzle.

## Interpretation of findings

The Wood review presented a synthesis of QBEs across a wider range of behaviors and study designs, and found a larger overall effect. Moderator analyses suggest that the QBE were larger studies conducted in laboratories and with student samples. Thus, an alternative conclusion that could be drawn from these findings is that the QBE might be an important source of error for research, especially in studies of students undertaken in laboratories. Despite significant heterogeneity and uncertainty, the Wood review makes strong recommendations for practice based on their findings, i.e., highlighting that the QBE may be “hugely valuable in social policy and public health terms” (p. 20) and generalize these findings by stating the potential of QBE in being “effective in promoting a wide range of behaviors” (p. 20). Despite the fundamental differences in the reviews undertaken, given the risk of bias in primary studies and the evidence for publication bias, the evidence to date does not seem robust enough to provide implications for public health practice and policy.

In conclusion, the reviews differ in terms of: (a) the inclusion of non-randomized studies by the Wood review, (b) the behavioral classification used, and (c) the inclusion of a risk of bias assessment by the Rodrigues review. To decide whether QBE could be an effective public health intervention, the key comparison that needs to be empirically tested is: does distributing questionnaires result in greater behavior change than simply sending prompts or other reminders?

## Author contributions

AR, DF, and FS conceived the idea for the manuscript together. All authors contributed to the preparation of the manuscript and critically reviewed the manuscript.

## Funding

AR and FS are funded by Fuse, the Centre for Translational Research in Public Health, a UK Clinical Research Collaboration Public Health Research Centre of Excellence based on funding from the British Heart Foundation, Cancer Research UK, Economic and Social Research Council, Medical Research.

### Conflict of interest statement

The authors declare that the research was conducted in the absence of any commercial or financial relationships that could be construed as a potential conflict of interest.

## Footnotes

^1^Fifteen of the randomized studies included in the Rodrigues review were not included in the Wood review.
